# Emergence of Highly Multidrug-Resistant Bacteria Isolated from Patients with Infections Admitted to Public Hospitals in Southwest Iran

**DOI:** 10.1155/2021/5265379

**Published:** 2021-08-13

**Authors:** Sepide Namdari, Ali Farhadi, Aida Khademalhoseini, Abbas Behzad-Behbahani, Afsaneh Moaddeb

**Affiliations:** ^1^Diagnostic Laboratory Sciences and Technology Research Center, School of Paramedical Sciences, Shiraz University of Medical Sciences, Shiraz, Iran; ^2^Qaem Hospital, Firuzabad, Fars, Iran; ^3^Department of Bacteriology and Virology, School of Medicine, Shiraz University of Medical Sciences, Shiraz, Iran

## Abstract

**Background:**

The emergence of multidrug-resistant (MDR) microorganisms causing infections is increasing worldwide and becoming more serious in developing countries. Among those, *Acinetobacter* species are becoming prominent.

**Objectives:**

The aim of this study was to determine the rate of antimicrobial resistance of the bacteria causing infections, *Acinetobacter s*pecies in particular, in local public hospitals in Firuzabad, Fars province, Iran.

**Methods:**

This cross-sectional study was performed on different clinical specimens collected from patients who were suspected of infections hospitalized from March 2016 to March 2019 in local hospitals of Firuzabad, Fars province, Iran. The bacterial isolates were identified following standard microbiological methods. Clinical and Laboratory Standards Institute guidelines were used to identify the antibiotic susceptibility of these isolates.

**Results:**

Overall, 1778 bacterial etiologies were isolated from 1533 patients diagnosed with infection. Of these, 1401 (78.8%) were Gram-negative and the remaining were Gram-positive bacteria. *Escherichia coli* (37.1%), *Klebsiella* spp. (13.9%), and *Acinetobacter* species (10.4%) were the most common isolated bacteria. Antibiotic sensitivity testing in this study showed a high resistance rate of *Acinetobacter* species to all antibiotics tested except Colistin. During the study period, the rate of infection with highly multidrug-resistant *Acinetobacter* species increased from 7.2% to 13.3%.

**Conclusions:**

This study highlights the emergence of MDR bacterial agents such as *Acinetobacter* species as a new threat in our region. However, a decrease in the rate of infection with *Pseudomonas aeruginosa* was noticeable.

## 1. Introduction

The emergence of antimicrobial-resistant bacteria in the community and hospitals is a critical threat to public health worldwide [1–3]. Unnecessary antibiotic use, excessive use of broad-spectrum antibiotics, and improper prescription of antibiotic drugs are the main reasons for the increased prevalence of antibiotic-resistant microorganisms [4]. Despite this rise in the prevalence of drug-resistant pathogens, the development of new antimicrobial agents is declining drastically [5]. Accordingly, the possibility of facing a rising number of potentially untreatable infections in the near future is a cause for concern. Furthermore, decreased sensitivity to the available antibiotics is a major concern in Iranian healthcare facilities [6–8].

A major challenge in treating patients with bacterial infections is the selection of appropriate antibiotics for their treatment. This can be obtained based on the information on the antimicrobial resistance patterns in the area. For this reason, updated data on antimicrobial resistance patterns in every region is required.

The goal of the present study was to provide up-to-date data on the antibiotic resistance patterns of bacterial infections in this area. Such data could provide a practical guide for physicians. More importantly, it would highlight the serious threat of multidrug-resistant (MDR) bacteria causing infections, some of which are entirely resistant to every antibiotic available. Studies like this will draw special attention to the necessity of future studies in order to find new medications for treating infections with such bacteria.

## 2. Methods

### 2.1. Study Subjects

This cross-sectional study was conducted within a 3-year period from March 2016 to March 2019 in local public hospitals in Firuzabad, southwest Iran. The samples were taken as a part of the routine diagnostic practice; however, after the approval of the ethics committee (Approval ID: IR.SUMS.REC.1393.8313), informed consent was obtained from each participant or legal guardian.

Using sterile equipment and aseptic techniques, 1778 clinical samples including blood, urine, sputum, wound swab, and endotracheal tube specimens (ETT) were collected from 1533 patients diagnosed with infection based on clinical signs and laboratory investigations. Patients were aged between 1 and 90 years old (38.7 ± 24.4 years). The specimens were taken from patients by medical nurses and laboratory technicians and were transported to the laboratory immediately for further analysis.

### 2.2. Sample Collection

For blood culture collection, the venipuncture method was used to obtain blood samples. Two sets of blood specimens were collected from different venipuncture sites. Each bottle consisted of 7–10 mL of blood for adult patients [9]. The collected volume of blood for pediatric patients was based on the weight of the patients [10].

For the diagnosis of urinary tract infections (UTI), clean-catch midstream urine (MSU), neonatal bagged urine, indwelling catheter (Foley catheter) urine, or suprapubic catheter urine was collected from patients.

The sputum samples were taken into sterile containers and were immediately analyzed microscopically by Gram staining. The samples containing less than 10 epithelial cells and more than 25 leukocytes in each area upon 100x magnification were included in the study as eligible sputum specimens [11].

ETTs were obtained immediately after extubation. Roughly, 1 cm of the distal end of the ETTs was cut for microbiological culture analysis. The tips were placed in a 15 mL conical tube containing 5 mL of sterile phosphate-buffered saline (PBS). Conical tubes were centrifuged at 400 g upon delivery, and pellets were used for further analysis.

For wound culture, wounds were first rinsed thoroughly with sterile saline solution. A small area (1 cm) of clean viable tissue was identified, and the sterile swab was rotated on it for 5 seconds while applying enough pressure to produce exudate. Swabs were then transferred into sterile containers. All the specimens were processed in the laboratory immediately (within 1 hour) to keep the samples stable.

### 2.3. Bacteriological Investigation

Culturing and identification of isolates were on the basis of standard guidelines for microbiological examination [12, 13]. Briefly, blood samples were collected as soon as the onset of clinical symptoms before administration of antimicrobial therapy. For the identification of pathogens, BACTEC™ (Becton Dickinson, USA) blood culture bottle system was employed. Blood culture specimens were incubated for 7 days. Positive blood cultures were plated on Columbia blood agar with 5% sheep blood, MacConkey agar, and chocolate agar. Blood and MacConkey agar plates were incubated for 2 days in an atmosphere with 5% CO_2_. Chocolate agar plates were incubated anaerobically in Gas-pack anaerobic jars with Gas-Pack envelopes (BBL; Becton Dickinson & Co., Cockeysville, Md., USA) and palladium catalyst to achieve and maintain an anaerobic atmosphere enriched with CO2.

Urine specimens were cultured on blood agar and MacConkey agar plates using calibrated 0.001 mL loops for quantitative urine cultures. Greater than or equal to 100 000 colony-forming units (CFU) of bacteria per mL of MSU or neonatal bagged urine samples were considered positive for infection. A positive growth of bacteria for other types of urine specimens was considered infection as well.

Respiratory specimens were routinely cultured onto several solid media, including chocolate agar, sheep blood agar, and MacConkey agar. Sputum cultures with more than 5 colonies per plate of potential respiratory pathogens were considered positive for infection.

The assessment of wound infection was performed by inoculating the swabs on blood agar, MacConkey agar, and chocolate agar and incubating at 37°C for 24 to 48 hours.

All culture media were supplied from bioMérieux, France. Bacterial isolates were identified using conventional methods based on their morphological, biochemical, and physiological characteristics. Briefly, Gram staining was performed on smears of inoculums of single colonies from pure subcultures in 20 *μ*l of sterile PBS. The stained slides were analyzed using light microscopy. Subsequently, the identification of the isolated Gram-positive and Gram-negative bacteria was carried out using biochemical tests. For the Gram-positive isolates, catalase, coagulase, DNase, Bacitracin, Novobiocin and Optochin susceptibility, hippurate hydrolysis, 6.5% NaCl broth salt tolerance, and bile esculin tests were applied. The identification of Gram-negative bacteria involved performing triple sugar iron (TSI), Simmon's citrate, sulfide-indole-motility (SIM), urease, methyl red (MR), Voges–Proskauer (VP), lysine decarboxylase, arginine decarboxylase, ornithine decarboxylase, phenylalanine deaminase, oxidase, oxidation-fermentation (OF), and acetate utilization tests [14].

### 2.4. Antibiotic Susceptibility Testing

The antimicrobial susceptibility test (AST) was performed for all isolates using the standard Kirby–Bauer disk diffusion method [15]. The antibiotics for disc diffusion testing for both Gram-positive and Gram-negative isolates were in the following concentrations: Ceftizoxime (30 *μ*g), Amikacin (30 *μ*g), Cefixime (5 *μ*g), Ciprofloxacin (5 *μ*g), Nitrofurantoin (200 *μ*g), Gentamicin (10 *μ*g), Nalidixic acid (30 *μ*g), Ceftriaxone (30 *μ*g), Co-Trimoxazole (25 *μ*g), Norfloxacin (10 *μ*g), Tetracycline (30 *μ*g), Chloramphenicol (30 *μ*g), and Imipenem (10 *μ*g). In case of some Gram-positive isolates, Vancomycin (30 *μ*g), Ampicillin (10 *μ*g), Clindamycin (2 *μ*g), and Erythromycin (15 *μ*g) were also used. Colistin in a concentration of 10 *μ*g was used for MDR pathogens such as *Acinetobacter* spp., *Pseudomonas aeruginosa*, and *Klebsiella pneumoniae*. MDR was defined as resistance to at least one antibiotic in at least three antimicrobial categories [16].

Antimicrobial discs were obtained from Padtan-TEB Co., Tehran, Iran. For the interpretation of antibiotic susceptibility testing, diameters of inhibition zones around the discs were measured and were classified as sensitive (S), intermediate (I), and resistant (R) as suggested by Clinical and Laboratory Standards Institute (CLSI) (https://clsi.org).

The quality control strains used in the study were *Escherichia coli* (ATCC®25922™), *Pseudomonas aeruginosa* (ATCC® 27853™), *Staphylococcus aureus* (ATCC®25923™), and *Enterococcus faecalis* (ATCC® 29212™).

### 2.5. Statistical Analysis

Data management and analysis was carried out using WHONET 5.6 software.

## 3. Results

Overall, 1533 patients developed infections during this three-year study. Among them, 889 (58%) were female and 644 (42%) were male. Infections with Gram-positive and Gram-negative bacteria were detected in 293 out of 1533 (19.1%) and in 1181 out of 1533 (77.0%) patients, respectively. Furthermore, coinfection with both Gram-positive and Gram-negative bacteria was found in 59 out of 1533 patients (3.8%). The distribution of bacterial isolates in different clinical specimens and hospital wards is shown in Tables 1 and 2, respectively.

**Figure 1:**
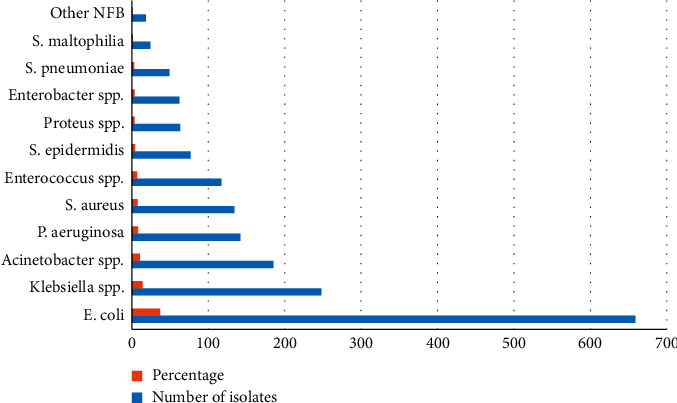
Frequency of bacteria isolated from clinical samples of patients with infections; NFB: nonfermentative bacteria.

Among Gram-negative bacteria, *E. coli* (37.1%)*, Klebsiella* spp. (13.9%), and *Acinetobacter* spp. (10.4%) were dominant causes of infections. However, *S. aureus* was the most prevalent isolate among Gram-positive bacteria (Figure 1).

The prevalence of bacteria involved in infections during the study period is presented in Table 3. There was a decrease in the rate of *Pseudomonas aeruginosa* infections from 12.2% in the years 2016-2017 to 4.8% in the years 2018-2019. However, an increasing trend in infections due to *Acinetobacter* spp. from 7.2% in the years 2016-2017 to 13.3% in the years 2018-2019 was demonstrated (Table 3). The antibiotic resistance patterns of the Gram-positive and Gram-negative bacteria are presented in Tables 4 and 5, respectively.

MDR patterns of Gram-positive and Gram-negative bacterial agents are shown in Tables 6 and 7 . The highest rate of multidrug resistance among Gram-positive bacteria was found in the isolates of *Enterococcus* spp. (91.4%), followed by *S. epidermidis* (64.9%) and *S. aureus* (38.8%), while among Gram-negative bacteria, the highest rate of multidrug resistance was detected in the isolates of *Acinetobacter* spp. (100%), followed by *Klebsiella* spp. (58.5%) and *E. coli* (54.0%).

Urinary tract infection was present in 62.1% of the patients, followed by respiratory tract infection (19.6%) and wound infection (15.5%). UTIs were more frequent among women (74%), and *E. coli* was the major cause of them (62.0%).

About fifty-five percent of the patients with respiratory tract infections were those who were receiving tracheal intubation, most of whom were hospitalized in intensive care units (ICU). The main bacteria isolated from ETT cultures were *Acinetobacter* spp. (44.9%). The same bacteria were the most frequent cause of positive sputum cultures (28.2%). These bacteria were highly resistant to most of the antibiotics. However, Colistin was the only antibiotic that all of the mentioned bacteria were still sensitive to.

*Staphylococcus epidermidis* was the major cause of positive blood cultures (20.0%) and these bacteria showed the most sensitivity to Vancomycin and were most resistant to Erythromycin. Furthermore, *Staphylococcus aureus* was the most frequent microorganism isolated from wound cultures (21.8%), all of which were sensitive to Vancomycin.

## 4. Discussion

In the present study, the pattern of antibiotic resistance of bacteria isolated from patients with infection was investigated. During three years of study, 1533 patients developed infections. When the incidence of infections was examined in different wards of general hospitals in Firuzabad, Fars province, Iran, the patients admitted in the internal medicine ward, in particular, had the highest rate of infection (28.9%) followed by surgical (orthopedic and general surgery) wards (23%), the pediatric ward (13%), neonatal ICU (12%), ICU (11.5%), CCU (7.4%), and emergency department (4.2%). UTI was the most frequent infection during the study period. *Escherichia coli* was found to be the most common pathogen isolated (37.1%), followed by *Klebsiella* spp. (13.9%) and *Acinetobacter* spp. (10.4%).

Overall, the prevalence of infections with Gram-negative bacteria was higher than Gram-positive bacteria (78.8% versus 21.2%). The most frequent Gram-negative bacteria causing infections were *E. coli* (21.6%), while in other studies *Enterobacter* spp., *Klebsiella pneumoniae,* and *Pseudomonas aeruginosa* were the most prevalent causative organisms of infections [17], the incidence of infection with *E. coli* and *Acinetobacter* spp. is increasing in our region. The prevalence of MDR among *Acinetobacter* spp. isolates was found to be 100%, which is far higher than reports from Saudi Arabia (74%) and Ethiopia (71.6%) [18, 19]. The emergence of MDR *Acinetobacter* spp. may complicate the choice of the accurate antibiotic for treatment and increase the mortality rate in hospitalized patients [20]. Except for Colistin (Polymyxin E) with 100% sensitivity, *Acinetobacter* spp. isolates exhibited high rates of resistance to all the antibiotics that are routinely used in clinical pathology laboratories for Gram-negative bacteria. These results contradict with a previous study in which drug resistance to Colistin was high (49.8%) in the northern part of Iran [21].

Furthermore, between 5.3% and 69.8% of isolated *E. coli* were resistant to different antibiotics. Among the tested antibiotics for *E. coli* isolates, the lowest antibiotic resistance was detected for Amikacin, followed by Colistin. However, these isolates were highly resistant to Nalidixic acid and Co-Trimoxazole, which is consistent with a previous report from Isfahan, Iran [22]. *Pseudomonas aeruginosa* isolates showed high rates of sensitivity to the studied antibiotics and were detected in only 8.0% of infections, which is far lower than infections with *E. coli* and *Acinetobacter* spp. isolated from clinical samples. The rate of MDR among *Pseudomonas aeruginosa* isolates was 9.8%, which is lower than the MDR rate of 31% reported by a recent study in Tehran, Iran [23].

*Staphylococcus aureus* was the most common Gram-positive bacteria isolated from infected patients and generally comprised 7.5% of all bacterial infections and 35.5% of all Gram-positive bacterial infections. *Staphylococcus aureus* was mostly isolated from patients with wound and respiratory tract infections. We did not find Vancomycin-resistant *Staphylococcus aureus* during the study period among infected patients. This finding is in line with that of a previous study on *Staphylococcus aureus* in which although all studied isolates were MDR, they were generally susceptible to Vancomycin [24].

*Enterococcus* spp., as the second most frequent isolated Gram-positive bacteria, showed a notably high multidrug resistance rate of 91.4%, which is comparable to the rates reported by a study in Taiwan [25]. Further, 75.2% of *Enterococcus* spp. isolates were found to be Vancomycin-resistant, which might be associated with the extensive use of Vancomycin in the hospital environment. Vancomycin-resistant Enterococci (VRE) have become a serious problem in almost every hospital and especially in high-risk patients [26–28].

There were 116 patients with the same bacterial isolates from different clinical samples. All isolates were tested for their antibiotic susceptibility. Approximately 80% of them had the similar patterns of antibiotic susceptibility. However, 52 patients had different bacterial species in clinical samples collected from multiple anatomical sites of infection.

## 5. Conclusion

In conclusion, the results indicate the increasing prevalence of infections with emerging opportunistic pathogens such as *Acinetobacter* spp. in our region. They are able to cause different types of infections. While the rate of infection with other Gram-positive and Gram-negative bacteria remains unchanged during the study period, a reduction in the rate of infection with *Pseudomonas aeruginosa* is evident. However, the emergence of MDR *Acinetobacter* spp. seems to become a major threat in the near future. Further, the considerable rate of infection with *E. coli* should not be ignored. Moreover, the molecular analysis of the isolates is recommended to characterize the antibiotic resistance genes.

## Figures and Tables

**Table 1:** Distribution of bacteria isolated from different samples collected from patients with infections.

Microorganism	Source of specimen					
	Total	Urine	Blood	Wound	Sputum	ETT
	Number of isolates (%)					
*Acinetobacter* spp.	185 (10.4)	0	9 (6.9)	19 (8.0)	82 (28.2)	75 (44.9)
*Enterobacter* spp.	62 (3.5)	44 (4.6)	6 (4.6)	12 (5.0)	0	0
*Enterococcus* spp.	117 (6.6)	77 (8.1)	12 (9.2)	28 (11.8)	0	0
*Escherichia coli*	659 (37.1)	590 (62.0)	21 (16.1)	15 (6.3)	22 (7.6)	11 (6.6)
*Klebsiella* spp.	248 (13.9)	154 (16.2)	13 (10.0)	27 (11.3)	34 (11.7)	20 (12.0)
*Proteus* spp.	63 (3.5)	41 (4.3)	5 (3.8)	17 (7.1)	0	0
*Pseudomonas aeruginosa*	142 (8.0)	29 (3.0)	8 (6.1)	39 (16.4)	35 (12.0)	31 (18.6)
*Staphylococcus aureus*	134 (7.5)	17 (1.8)	12 (9.2)	52 (21.8)	34 (11.7)	19 (11.4)
*Staphylococcus epidermidis*	77 (4.3)	0	26 (20.0)	29 (12.2)	11 (3.8)	11 (6.6)
*Stenotrophomonas maltophilia*	24 (1.3)	0	11 (8.5)	0	13 (4.5)	0
*Streptococcus pneumoniae*	49 (2.7)	0	0	0	49 (16.8)	0
Other nonfermentative bacteria	18 (1.0)	0	7 (5.4)	0	11 (3.8)	0
Total	1778	952	130	238	291	167

ETT: endotracheal tube specimen.

**Table 2:** Distribution of bacteria isolated from patients with infections in different hospital wards.

Microorganism	Hospital ward						
	Internal medicine	Surgical wards	Pediatric ward	NICU	ICU	CCU	Emergency department
	Number of isolates (%)						
*Acinetobacter* spp.	25 (4.9)	29 (7.1)	19 (8.2)	32 (15.0)	59 (28.9)	18 (13.6)	3 (4.0)
*Enterobacter* spp.	18 (3.5)	16 (3.9)	5 (2.2)	2 (0.9)	6 (2.9)	12 (9.1)	3 (4.0)
*Enterococcus* spp.	10 (1.9)	38 (9.3)	7 (3.0)	13 (6.1)	25 (12.2)	18 (13.6)	6 (8.0)
*Escherichia coli*	265 (51.6)	168 (41.1)	105 (45.4)	49 (23.0)	30 (14.7)	13 (9.8)	29 (38.7)
*Klebsiella* spp.	94 (18.3)	22 (5.4)	35 (15.1)	38 (17.8)	15 (7.3)	26 (19.7)	18 (24)
*Proteus* spp.	19 (3.7)	18 (4.4)	4 (1.7)	7 (3.3)	5 (2.4)	6 (4.5)	4 (5.3)
*Pseudomonas aeruginosa*	19 (3.7)	26 (6.4)	15 (6.5)	34 (15.9)	27 (13.2)	19 (14.4)	2 (2.7)
*Staphylococcus aureus*	34 (6.6)	62 (15.2)	6 (2.6)	13 (6.1)	9 (4.4)	8 (6.1)	2 (2.7)
*Staphylococcus epidermidis*	14 (2.7)	16 (3.9)	12 (5.2)	8 (3.7)	18 (8.8)	6 (4.5)	3 (4.0)
*Stenotrophomonas maltophilia*	3 (0.6)	7 (1.7)	5 (2.2)	3 (1.4)	3 (1.5)	2 (1.5)	1 (1.3)
*Streptococcus pneumoniae*	9 (1.7)	0	17 (7.4)	12 (5.6)	5 (2.4)	3 (2.3)	3 (4.0)
Other nonfermentative bacteria	4 (0.8)	7 (1.7)	1 (0.4)	2 (0.9)	2 (1.0)	1 (0.8)	1 (1.3)
Total	514	409	231	213	204	132	75

**Table 3:** Prevalence of bacterial infections during the three-year study period.

Microorganism	Sample collection dates		
	2016-2017	2017-2018	2018-2019
	Number of isolates (%)		
*Acinetobacter* spp.	39 (7.2)	58(10.0)	88 (13.3)
*Enterobacter* spp.	17 (3.1)	23 (4.0)	22 (3.3)
*Enterococcus* spp.	43 (8.0)	39 (6.7)	35 (5.3)
*Escherichia coli*	188 (34.9)	217 (37.3)	254 (38.5)
*Klebsiella* spp.	69 (12.8)	83 (14.3)	96 (14.6)
*Proteus* spp.	27 (5.0)	22 (3.8)	14 (2.1)
*Pseudomonas aeruginosa*	66 (12.2)	44 (7.6)	32 (4.8)
*Staphylococcus aureus*	45 (8.4)	44 (7.6)	45 (6.8)
*Staphylococcus epidermidis*	23 (4.3)	19 (3.3)	35 (5.3)
*Stenotrophomonas maltophilia*	5 (0.9)	10 (1.7)	9 (1.4)
*Streptococcus pneumoniae*	12 (2.2)	15 (2.6)	22 (3.3)
Other nonfermentative bacteria	4 (0.7)	7 (1.2)	7 (1.1)
Total	538	581	659

**Table 4:** Antibiotic resistance patterns of Gram-positive bacteria isolated from patients with infections.

Antibiotics	*Enterococcus spp.*			*S. aureus*			*S. epidermidis*			*S. pneumoniae*		
	R%	I%	S%	R%	I%	S%	R%	I%	S%	R%	I%	S%
Amikacin	NA	NA	NA	3.0	0	97.0	7.8	3.9	88.3	NA	NA	NA
Ciprofloxacin	93.2	5.1	1.7	22.4	4.5	73.1	63.6	1.3	35.1	NA	NA	NA
Nitrofurantoin	11.1	21.4	67.5	20.1	7.5	72.4	3.9	3.9	92.2	NA	NA	NA
Gentamicin	84.6	8.5	6.8	14.2	0	85.8	28.6	5.2	66.2	NA	NA	NA
Ceftriaxone	NA	NA	NA	30.6	5.2	64.2	71.4	11.7	16.9	42.9	4.1	53.1
SXT	NA	NA	NA	20.1	3.0	76.9	58.4	1.3	40.2	32.6	4.1	63.3
Norfloxacin	94	4.3	1.7	39.6	6.7	53.7	63.6	1.3	35.1	NA	NA	NA
Tetracycline	92.3	0	7.7	42.5	0.7	56.7	64.9	0	35.1	51.0	6.1	42.9
Vancomycin	75.2	17.9	6.8	0	0	100	1.3	0	98.7	0	0	100
Ampicillin	60.7	4.3	35.0	NA	NA	NA	NA	NA	NA	NA	NA	NA
Chloramphenicol	32.5	13.7	53.8	8.2	6.0	85.8	5.2	3.9	90.9	12.2	0	87.7
Clindamycin	NA	NA	NA	33.6	4.5	61.9	71.4	2.6	26.0	28.6	2.0	69.4
Erythromycin	NA	NA	NA	38.8	5.2	56.0	84.4	0	15.6	30.6	6.1	63.2

R: resistant, I: intermediate, S: sensitive, SXT: Co-Trimoxazole, and NA: not applicable.

**Table 5:** Antibiotic resistance patterns of Gram-negative bacteria isolated from patients with infections.

Antibiotics	*E. coli*			*Klebsiella* spp.			*Enterobacter* spp.			*P. aeruginosa*			*Acinetobacter* spp.			*S. maltophilia*			Other NFB			*Proteus spp.*		
	R%	I%	S%	R%	I%	S%	R%	I%	S%	R%	I%	S%	R%	I%	S%	R%	I%	S%	R%	I%	S%	R%	I%	S%
Ceftizoxime	53.6	8.6	37.8	52.0	2.8	45.2	37.1	6.4	56.4	NA	NA	NA	NA	NA	NA	NA	NA	NA	NA	NA	NA	11.1	1.6	87.3
Amikacin	5.3	5	89.6	22.6	2.4	75.0	8.0	6.4	85.5	9.9	1.4	88.7	95.7	2.7	1.6	8.3	0	91.7	44.4	0	55.6	0	0	100
Cefixime	63.7	1.4	34.9	54.4	2.0	43.5	48.4	4.8	46.8	NA	NA	NA	NA	NA	NA	NA	NA	NA	NA	NA	NA	12.7	4.8	82.5
Ciprofloxacin	54.9	6.4	38.7	30.6	6.8	62.5	14.5	4.8	80.6	15.5	0	84.5	98.9	0.5	0.5	4.2	0	95.8	11.1	22.2	66.7	28.6	6.3	65.1
Nitrofurantoin	18.7	12.0	69.2	68.5	23.0	8.5	69.3	6.4	24.2	NA	NA	NA	NA	NA	NA	NA	NA	NA	NA	NA	NA	NA	NA	NA
Gentamicin	27.0	0.9	72.1	28.2	0.4	71.3	21.0	0	79.0	9.9	0.7	89.4	94.6	1.1	4.3	8.3	0	91.7	44.4	0	55.6	6.3	0	93.6
Nalidixic acid	69.8	2.7	27.5	35.1	10.1	54.8	35.5	8.0	56.4	NA	NA	NA	NA	NA	NA	NA	NA	NA	NA	NA	NA	52.4	0	47.6
Ceftriaxone	64.0	0.9	35	37.1	5.6	57.3	32.3	6.4	61.3	NA	NA	NA	69.7	1.6	28.6	NA	NA	NA	NA	NA	NA	7.9	11.1	80.9
SXT	66.2	0.3	33.5	47.2	4.0	48.8	30.6	3.2	66.1	NA	NA	NA	96.8	1.6	1.6	8.3	0	91.7	27.8	0	72.2	65.1	0	34.9
Norfloxacin	56.8	3.2	40	27.8	1.2	71.0	21.0	0	79.0	27.5	0	72.5	NA	NA	NA	NA	NA	NA	NA	NA	NA	23.8	0	76.2
Tetracycline	63.7	0.6	35.7	38.3	12.1	49.6	33.9	9.7	56.4	NA	NA	NA	73.0	3.2	23.8	16.7	0	83.3	55.6	5.6	38.9	NA	NA	NA
Chloramphenicol	24.7	8.6	66.6	32.7	4.0	63.3	24.2	9.7	66.1	NA	NA	NA	NA	NA	NA	0	8.3	91.7	61.1	22.2	16.7	41.3	17.5	41.3
Imipenem	41.0	20.0	39.0	40.3	16.9	42.7	25.8	8.0	66.1	15.5	9.1	75.3	99.5	0	0.5	66.7	8.3	25.0	50.0	16.7	33.3	NA	NA	NA
Colistin	6.8	0	93.2	1.2	0	98.8	6.4	0	93.5	0	0	100	0	0	100	12.5	0	87.5	44.4	0	55.6	NA	NA	NA

R: resistant, I: intermediate, S: sensitive, SXT: Co-Trimoxazole, NFB: nonfermentative bacteria, and NA: not applicable.

**Table 6:** Multidrug resistance patterns of Gram-positive bacteria isolated from patients with infections.

Microorganism	Number of antibiotics	Antibiotic resistant isolates No (%)	Multidrug resistance patterns
*Enterococcus* spp. (No = 117)	5	48 (41.0)	CIP, TE, GM, V, AMP
	4	26 (22.2)	CIP, TE, GM, V
	4	19 (16.2)	CIP, TE, GM, AMP
	3	6 (5.1)	CIP, TE, GM
	3	8 (6.8)	CIP, TE, V

*S. aureus* (No = 134)	5	3 (2.2)	CIP, GM, E, CC, SXT
	4	10 (7.5)	CIP, E, CC, TE
	3	6 (4.5)	E, SXT, CHL
	3	3 (2.2)	GM, E, CHL
	3	13 (9.7)	E, CC, TE
	3	8 (6.0)	CIP, E, CC
	3	9 (6.7)	CIP, GM, E

*S. epidermidis* (No = 77)	5	5 (6.5)	CIP, GM, E, CC, SXT
	4	6 (7.8)	E, CC, TE, SXT
	4	5 (6.5)	CIP, E, CC, TE
	3	2 (2.6)	GM, E, CHL
	3	8 (10.4)	E, CC, TE
	3	11 (14.3)	CIP, E, CC
	3	13 (16.9)	E, TE, SXT

*S. pneumoniae* (No = 49)	4	3 (6.1)	E, CC, TE, SXT
	3	5 (10.2)	E, CC, TE

CIP: Ciprofloxacin, TE: Tetracycline, GM: Gentamicin, V: Vancomycin, AMP: Ampicillin, E: Erythromycin, CC: Clindamycin, SXT: Co-Trimoxazole, and CHL: Chloramphenicol.

**Table 7:** Multidrug resistance patterns of Gram-negative bacteria isolated from patients with infections.

Microorganism	Number of antibiotics	Antibiotic resistant isolates No (%)	Multidrug resistance patterns
*Escherichia coli* (No = 659)	6	45 (6.8)	SXT, TE, IMP, GM, CHL, COL
	5	35 (5.3)	SXT, CRO, CIP, GM, AMK
	4	58 (8.8)	SXT, TE, CIP, GM
	4	36 (5.5)	SXT, TE, CHL, CIP
	3	62 (9.4)	SXT, TE, CHL
	3	66 (10)	SXT, TE, IMP
	3	54 (8.2)	SXT, CRO, CIP

*Klebsiella* spp. (No = 248)	5	3 (1.2)	SXT. TE, CIP, GM, COL
	4	47 (18.9)	CRO, CIP, GM, AMK
	3	33 (13.3)	SXT, TE, IMP
	3	36 (14.5)	SXT, TE, CHL
	3	26 (10.5)	SXT, CRO, CIP

*Enterobacter* spp. (No = 62)	4	4 (6.4)	CRO, CIP, GM, AMK
	3	6 (9.7)	SXT, TE, CRO
	3	7 (11.3)	SXT, TE, IMP
	3	6 (9.7)	SXT, TE, CHL

*P. aeruginosa* (No = 142)	4	14 (9.9)	IMP, CIP, GM, AMK

*Acinetobacter* spp. (No = 185)	7	129 (69.7)	SXT, TE, IMP, CRO, CIP, GM, AMK
	6	6 (3.2)	SXT, TE, IMP, CIP, GM, AMK
	5	31 (16.7)	SXT, IMP, CIP, GM, AMK
	4	6 (3.2)	IMP, CIP, GM, AMK
	4	2 (1.1)	SXT, IMP, GM, AMK
	4	1 (0.5)	SXT, CIP, GM, AMK
	4	2 (1.1)	SXT, IMP, CIP, AMK
	3	8 (4.3)	SXT, IMP, CIP

*Proteus* spp. (No = 63)	3	5 (7.9)	SXT, CHL, CIP

SXT: Co-Trimoxazole, TE: Tetracycline, IMP: Imipenem, GM: Gentamicin, CHL: Chloramphenicol, COL: Colistin, CRO: Ceftriaxone, CIP: Ciprofloxacin, and AMK: Amikacin.

## Data Availability

Data are available within the article.

## References

[1] Roca I., Akova M., Baquero F. (2015). The global threat of antimicrobial resistance: science for intervention. *New Microbes and New Infections*.

[2] Lawton R. (1999). Intensive care antimicrobial resistance epidemiology (ICARE) surveillance report, data summary from January 1996 through December 1997. *American Journal of Infection Control*.

[3] Bradley J. S., Guidos R., Baragona S. (2007). Anti-infective research and development-problems, challenges, and solutions. *The Lancet Infectious Diseases*.

[4] Okeke I. N., Lamikanra A., Edelman R. (1999). Socioeconomic and behavioral factors leading to acquired bacterial resistance to antibiotics in developing countries. *Emerging Infectious Diseases*.

[5] Spellberg B., Guidos R., Gilbert D. (2008). The epidemic of antibiotic-resistant infections: a call to action for the medical community from the Infectious Diseases Society of America. *Clinical Infectious Diseases*.

[6] Jahansepas A., Aghazadeh M., Rezaee M. A. (2018). Occurrence of Enterococcus faecalis and Enterococcus faeciumin various clinical infections: detection of their drug resistance and virulence determinants. *Microbial Drug Resistance*.

[7] Razavi Nikoo H., Ardebili A., Mardaneh J. (2017). Systematic review of antimicrobial resistance of ClinicalAcinetobacter baumanniiIsolates in Iran: an update. *Microbial Drug Resistance*.

[8] Poorabbas B., Mardaneh J., Rezaei Z. (2015). Nosocomial Infections: multicenter surveillance of antimicrobial resistance profile of *Staphylococcus aureus* and Gram negative rods isolated from blood and other sterile body fluids in Iran. *Iranian Journal of Microbiology*.

[9] Kirn T. J., Weinstein M. P. (2013). Update on blood cultures: how to obtain, process, report, and interpret. *Clinical Microbiology and Infection*.

[10] Kellogg J. A., Manzella J. P., Bankert D. A. (2000). Frequency of low-level bacteremia in children from birth to fifteen years of age. *Journal of Clinical Microbiology*.

[11] Aydemir O., Aydemir Y., Ozdemir M. (2014). The role of multiplex PCR test in identification of bacterial pathogens in lower respiratory tract infections. *Pakistan Journal of Medical Sciences*.

[12] Winn W. (2006). Introduction to microbiology part II: guidelines for the collection, transport, processing, analysis and reporting of cultures from specific specimen sources. *Koneman’s Color Atlas and Textbook of Diagnostic Microbiology*.

[13] Collee J. G., Miles R., Watt B. (1996). Tests for identification of bacteria. *Mackie and McCartney Practical Medical Microbiology*.

[14] Holt J. G., Krieg N. R., Sneath P. H. (2013). *Bergey’s Manual of Determinative Bacterology*.

[15] Wayne P. (2011). *Performance Standards for Antimicrobial Susceptibility Testing*.

[16] Magiorakos A.-P., Srinivasan A., Carey R. B. (2012). Multidrug-resistant, extensively drug-resistant and pandrug-resistant bacteria: an international expert proposal for interim standard definitions for acquired resistance. *Clinical Microbiology and Infection*.

[17] Elliott C., Justiz-Vaillant A. (2018). Nosocomial infections: a 360-degree review. *International Biological and Biomedical Journal*.

[18] Almaghrabi M. K. (2018). Multidrug-resistant Acinetobacter baumannii: an emerging health threat in aseer region, Kingdom of Saudi Arabia. *Canadian Journal of Infectious Diseases and Medical Microbiology*.

[19] Ayenew Z., Tigabu E., Syoum E., Ebrahim S., Assefa D., Tsige E. (2021). Multidrug resistance pattern of Acinetobacter species isolated from clinical specimens referred to the Ethiopian Public Health Institute: 2014 to 2018 trend anaylsis. *PLoS One*.

[20] Gorbich Y., Karpov I., Kretchikova O. (2013). Impact of appropriate antimicrobial therapy on survival in patients with Acinetobacter baumannii-associated infections. *Journal of Microbiology and Infectious Diseases*.

[21] Amini M. (2018). Pattern of antibiotic resistance in nosocomial infections with Gram-negative bacilli in ICU patients (Tehran, Iran) during the years 2012-2014. *Journal of Basic and Clinical Pathophysiology*.

[22] Dehbanipour R., Rastaghi S., Sedighi M., Maleki N., Faghri J. (2016). High prevalence of multidrug-resistance uropathogenic *Escherichia coli* strains, Isfahan, Iran. *Journal of Natural Science, Biology, and Medicine*.

[23] Davarzani F. (2021). Evaluation of antibiotic resistance pattern, alginate and biofilm production in clinical isolates of *Pseudomonas aeruginosa*. *Iranian Journal of Public Health*.

[24] Ghaznavi-Rad E., Neela V., Nor Shamsudin M. (2012). Diversity in the antimicrobial susceptibility patterns of methicillin-resistant *Staphylococcus aureus* clones. *European Journal of Clinical Microbiology & Infectious Diseases*.

[25] Wang J.-T., Chang S.-C., Wang H.-Y., Chen P.-C., Shiau Y.-R., Lauderdale T.-L. (2013). High rates of multidrug resistance in *Enterococcus faecalis* and E. faecium isolated from inpatients and outpatients in Taiwan. *Diagnostic Microbiology and Infectious Disease*.

[26] Linfield R. Y., Campeau S., Injean P. (2018). Practical methods for effective vancomycin-resistant enterococci (VRE) surveillance: experience in a liver transplant surgical intensive care unit. *Infection Control & Hospital Epidemiology*.

[27] Anderson D. J., Moehring R. W., Weber D. J. (2018). Effectiveness of targeted enhanced terminal room disinfection on hospital-wide acquisition and infection with multidrug-resistant organisms and *Clostridium difficile*: a secondary analysis of a multicentre cluster randomised controlled trial with crossover design (BETR Disinfection). *The Lancet Infectious Diseases*.

[28] Taneja N., Rani P., Emmanuel R., Sharma M. (2004). Significance of vancomycin resistant enterococci from urinary specimens at a tertiary care centre in northern India. *The Indian Journal of Medical Research*.

